# Argumentos de la industria alimentaria en contra del etiquetado frontal de advertencias nutricionales en Uruguay

**DOI:** 10.26633/RPSP.2020.20

**Published:** 2020-04-02

**Authors:** Gastón Ares, Isabel Bove, Rodrigo Díaz, Ximena Moratorio, Wilson Benia, Fabio Gomes

**Affiliations:** 1 Sensometría & Ciencia del Consumidor Instituto Polo Tecnológico de Pando Universidad de la República Canelones Uruguay Sensometría & Ciencia del Consumidor, Instituto Polo Tecnológico de Pando, Universidad de la República, Canelones, Uruguay.; 2 Ministerio de Salud Pública Ministerio de Salud Pública Uruguay Ministerio de Salud Pública, Uruguay.; 3 Organización Panamericana de la Salud Organización Panamericana de la Salud Montevideo Uruguay Organización Panamericana de la Salud, Montevideo, Uruguay.; 4 Organización Panamericana de la Salud Organización Panamericana de la Salud Washington D.C. Estados Unidos de América Organización Panamericana de la Salud, Washington D.C., Estados Unidos de América.

**Keywords:** Etiquetado nutricional, etiquetado de alimentos, política pública, programas y políticas de nutrición y alimentación, información nutricional, Uruguay, Nutritional labeling, food labeling, public policy, nutrition programs and policies, nutritional facts, Uruguay, Rotulagem nutricional, rotulagem de alimentos, política pública, programas e políticas de nutrição e alimentação, informação nutricional, Uruguai

## Abstract

**Objetivo.:**

Identificar los argumentos de la industria alimentaria en contra del etiquetado frontal de advertenciasnutricionales en Uruguay.

**Métodos.:**

Se realizó un análisis de contenido de los comentarios de la industria alimentaria recibidos durante la consulta pública implementada durante el proceso de diseño del decreto que reglamenta la inclusión de rotulación nutricional frontal basada en advertencias en Uruguay mediante una codificación inductiva.

**Resultados.:**

La mayoría de los comentarios manifestaron la preocupación por la elevada prevalencia de obesidad y enfermedades no transmisibles en el país y destacaron el compromiso con la implementación de acciones que contribuyan a combatir esta problemática de salud. Sin embargo, 81% de los comentarios planteó desacuerdo con la propuesta de decreto elevada a consulta pública y realizó críticas generales sobre su validez. El contenido de los comentarios se relacionó con siete dimensiones: falta de justificación de la medida, violación de compromisos internacionales, críticas al sistema de advertencias, discriminación a alimentos industrializados envasados, críticas al modelo de perfil de nutrientes, otras críticas a aspectos específicos del proyecto de decreto y efectos económicos negativos. En cada dimensión se discute la pertinencia y consistencia técnica y normativa de los contenidos vertidos por los representantes de la industria.

**Conclusiones.:**

Bajo un aparente acuerdo con la necesidad de adoptar medidas, se evidenció, sin embargo, una fuerte oposición a la política y en particular al sistema de advertencias por parte de la industria alimentaria. Los resultados del análisis aportan insumos para facilitar el proceso de implementación de regulaciones similares en otros países.

## Abstract (en)

**Objective.:**

To identify the food industry’s arguments against front-of-package nutrition labels in Uruguay.

**Methods.:**

Content analysis and inductive coding were applied to the comments made by the food industry during the public consultation on the draft decree regulating the inclusion of front-of-package nutrition labels (warnings) in Uruguay.

**Results.:**

Most of the comments expressed concern about the high prevalence of obesity and noncommunicable diseases in the country and emphasized a commitment to implementing actions to help combat these health problems. However, 81% of the comments disagreed with the draft decree presented for public consultation; these included general criticisms of its validity. The content of the comments related to seven areas: lack of rationale for the measure; violation of international commitments; criticisms of the warning system; discrimination against industrialized packaged food; criticisms of the nutrient profile model; other criticisms of aspects specific to the draft decree; and adverse economic effects. For each area, there is discussion of the technical and normative relevance and consistency of the content presented by the industry representatives.

**Conclusions.:**

Despite apparent agreement about the need to adopt measures, it is clear that the food industry is strongly opposed to the policy and, in particular, to the warning system. The results of this analysis may help to facilitate the process of implementing similar regulations in other countries.

## RESUMO

**Objetivo.:**

Identificar os argumentos da indústria alimentícia contra a rotulagem frontal de advertência nutricional no Uruguai.

**Métodos.:**

Realizou-se uma análise do conteúdo dos comentários de representantes da indústria alimentícia obtidos em uma consulta pública conduzida no Uruguai durante o processo de elaboração do decreto para regulamentar a inclusão de rotulagem frontal de advertência nutricional com uso de códigos indutivos.

**Resultados.:**

Os comentários, na sua maioria, revelaram preocupação com a alta prevalência de obesidade e doenças crônicas não transmissíveis no país e destacaram o compromisso com a implementação de ações para combater estes problemas de saúde. No entanto, verificou-se que, em 81% dos comentários, os participantes discordavam da proposta do decreto apresentada na consulta pública e teceram críticas gerais quanto à sua validade. Observaram-se sete dimensões distintas no conteúdo dos comentários: falta de justificação da medida, descumprimento de compromissos internacionais, críticas ao sistema de advertência, discriminação de alimentos industrializados em embalagens, críticas ao modelo de perfil nutricional, outras críticas relativas a aspectos específicos do projeto do decreto e repercussão econômica negativa. Para cada dimensão, foram discutidas a relevância e a coerência técnica e normativa do conteúdo dos comentários feitos pelos representantes da indústria.

**Conclusões.:**

Apesar de uma suposta concordância quanto à necessidade de adotar medidas, observou-se uma firme oposição da indústria alimentícia à política e, em particular, ao sistema de advertência nutricional propostos. Os resultados desta análise servem para facilitar o processo de implementação de regulamentações semelhantes em outros países.

La inclusión de etiquetado nutricional en los envases de alimentos preenvasados es una de las políticas recomendadas para promover dietas más saludables y combatir la epidemia de enfermedades no transmisibles a nivel mundial (1). De acuerdo con el *Codex Alimentarius* (2), el etiquetado nutricional es “toda descripción destinada a informar al consumidor sobre las propiedades nutricionales de un alimento”. El etiquetado nutricional comprende dos elementos: la declaración de nutrientes (es decir, la enumeración del contenido de nutrientes del alimento) y la información nutricional complementaria, destinada a facilitar el entendimiento de los consumidores del valor nutricional de un alimento.

La declaración de nutrientes no suele ser utilizada por los consumidores debido a que resulta difícil de encontrar y de entender (3). Por este motivo, se ha recomendado la utilización de etiquetado nutricional en el frente de los envases como parte de la información nutricional complementaria para promover decisiones informadas (4). La ubicación frontal de esta información podría incentivar a los consumidores a considerar las características nutricionales de los alimentos en el momento de realizar sus decisiones de compra y motivar así elecciones más saludables (5).

Uno de los sistemas de rotulación nutricional que ha ganado popularidad en las Américas son las advertencias nutricionales, las cuales identifican productos con contenido excesivo de nutrientes asociados con enfermedades no transmisibles (6). Este sistema es fácil de encontrar en las etiquetas y fácil de interpretar, y podría motivar la selección de alimentos más saludables (7-9).

Se ha acumulado evidencia de que las industrias de productos alimenticios procesados y ultraprocesados, así como las industrias tabacaleras y de bebidas alcohólicas, ejercen actividades políticas corporativas para impedir, retrasar o debilitar políticas de gobierno que podrían limitar su expansión y el aumento de la demanda de sus productos en el mercado (10, 11). Por lo tanto, no puede esperarse que las advertencias nutricionales sean fácilmente aceptadas por la industria alimentaria, quien ha demostrado una fuerte oposición hacia sistemas de etiquetado nutricional frontal que indiquen en forma explícita que un producto o sus ingredientes no son recomendados como parte de una alimentación saludable (12).

En este contexto, el presente trabajo tuvo como objetivo identificar los argumentos de la industria alimentaria en contra del etiquetado frontal de advertencias nutricionales en Uruguay.

## MATERIALES Y MÉTODOS

En el presente trabajo se analizaron los comentarios de la industria alimentaria recibidos durante la consulta pública realizada en el marco de la elaboración del decreto de rotulación nutricional frontal en Uruguay, el cual fue aprobado en agosto de 2018 (13). El decreto plantea que los alimentos envasados en ausencia del cliente deben incluir símbolos con diseño octagonal, fondo negro y borde blanco con la expresión “exceso” seguida del nutriente que corresponda (grasa, grasas saturadas, azúcares o sodio), siempre que en su proceso de elaboración, o en el de alguno de sus ingredientes, se haya agregado sodio, azúcares o grasas, y en cuya composición final el contenido de sodio, azúcares, grasas o grasas saturadas exceda los valores establecidos, los cuales se basan en el modelo de perfil de nutrientes de la Organización Panamericana de la Salud (14). De acuerdo con el decreto, el rotulado frontal tiene como objetivo permitirle al consumidor identificar los alimentos envasados con contenido excesivo de nutrientes asociados al aumento del sobrepeso, la obesidad y las enfermedades no transmisibles.

La consulta fue implementada por el Ministerio de Industria, Energía y Minería de Uruguay a través de su página web entre el 2 de junio y el 16 de agosto de 2017. En la página web antes mencionada se encontraba disponible el proyecto de decreto y la dirección de correo electrónico a la que toda persona o institución interesada podía enviar sus comentarios (15). En paralelo, el Ministerio de Relaciones Exteriores elevó el proyecto a la secretaría de la Organización Mundial de Comercio (OMC) para recibir comentarios y observaciones a nivel internacional.

El acceso a los comentarios recibidos en la consulta pública se obtuvo a través de una solicitud de acceso a la información en el marco de la Ley de acceso a la información pública (16). La investigación se realizó siguiendo los requerimientos éticos aplicables a este tipo de estudio, incluida la conservación del anonimato de los participantes.

Se recibieron un total de 140 comentarios a la consulta pública, 42 de los cuales procedieron de industrias y asociaciones industriales uruguayas y extranjeras. Se realizó un análisis de contenido de dichos comentarios mediante codificación inductiva (17). Tres investigadores identificaron categorías correspondientes a temas a medida que fueron apareciendo al leer los comentarios enviados. Cada investigador realizó la codificación de forma independiente y abierta en función de los datos y su bagaje teórico, y el conocimiento de conceptos trabajados en investigaciones previas. La discrepancia en la codificación realizada por los investigadores se resolvió por triangulación. Una vez identificadas las categorías, se determinó el porcentaje de los comentarios que incluyó menciones a cada una de ellas. Por último, se agruparon las categorías en dimensiones utilizando el mismo procedimiento.

## RESULTADOS Y DISCUSIÓN

La mayoría de los comentarios recibidos de la industria de alimentos provinieron de asociaciones industriales internacionales (52%), seguido por industrias uruguayas (29%), cámaras industriales uruguayas (10%) e industrias extranjeras (9%). En general, los comentarios manifestaron preocupación por la elevada prevalencia de obesidad y enfermedades no transmisibles en el país y destacaron el compromiso con la implementación de acciones que contribuyan a combatir esta problemática de salud. Sin embargo, 81% de los comentarios planteó desacuerdo con la propuesta de decreto y realizó críticas generales sobre su pertinencia y justificación. El resto de los comentarios tuvo como objetivo realizar consultas o consideraciones puntuales sobre el proyecto. El contenido de los comentarios estuvo vinculado con siete dimensiones (cuadro 1) que se presentan y discuten a continuación.

### Falta de justificación de la medida

La industria cuestionó la justificación de la medida con el argumento de que la inclusión del etiquetado nutricional frontal basado en advertencias nutricionales no contribuirá a reducir la prevalencia de obesidad y enfermedades no transmisibles. Sin embargo, el objetivo de la política planteado en el decreto es facilitar la identificación de alimentos envasados con excesivo contenido de azúcares, grasas, grasas saturadas y sodio. En este sentido, existe suficiente evidencia científica de que el etiquetado nutricional frontal basado en advertencias es adecuado para alcanzar el objetivo planteado (7-9).

Los comentarios también destacaron que el proyecto no consideraba los múltiples factores que determinan la obesidad y las enfermedades no transmisibles, desconociendo o ignorando que la rotulación frontal es solo una de las medidas recomendadas para combatir esta problemática de salud (1). De acuerdo con los comentarios, la promoción de hábitos alimentarios saludables debe realizarse a través de educación. Este argumento ha sido utilizado por la industria en diversos países donde se ha impulsado etiquetado nutricional frontal (18-20). Si bien la educación y la motivación individual son importantes para lograr cambios en los hábitos, es difícil que estos cambios se logren a nivel poblacional si no existe un ambiente que promueva y facilite elecciones saludables (18).

**CUADRO 1.** Dimensiones y categorías identificadas en el análisis de los comentarios de la industria de alimentos durante la consulta pública realizada en el marco del diseño del decreto uruguayo de etiquetado nutricional frontal basado en advertencias

Dimensión	Categoría	Menciones (%)
Falta de justificación de la medida	Falta de evidencia científica de que el decreto cumplirá con el objetivo	48
	No considera multicausalidad de obesidad y enfermedades no transmisibles	45
	Cambios en los hábitos se logran con educación	55
Violación de compromisos internacionales	Creación de obstáculos técnicos al comercio	62
	Incompatibilidad con normativa MERCOSUR	55
	Ausencia de normas *Codex*	12
Críticas al sistema de advertencias	Estigmatiza y genera miedo en el consumidor	62
	Énfasis en alimentos individuales como “negativos”	60
	Genera confusión	57
	Contraviene disposiciones del *Codex Alimentarius*	55
	Falta de justificación científica	50
	No se incluye información cuantitativa	24
	Utilización de otros sistemas	21
Discriminación a alimentos industrializados envasados	Restricción a alimentos preenvasados	52
	Enfoque negativo a alimentos industrializados	45
	Alimentos industrializados cumplen estándares de inocuidad y calidad	24
	Falta de evidencia basada en estadísticas de consumo	12
Críticas al modelo de perfil de nutrientes	Falta de validez del modelo de perfil de nutrientes de OPS	62
	No se estimula reformulación	62
	Elevado porcentaje de alimentos llevarían advertencias	43
	No considera porción de consumo	26
	No considera nutrientes intrínsecos	26
	Modificación de límite de sodio en productos bajos en calorías	14
	Contenido de nutrientes debería expresarse por cada 100 g o porción	12
	Límites deben establecerse por categoría	12
	Excluir grasas totales	10
	Excluir nutrientes intrínsecos	10
	Incluir calorías	4
Otras críticas a aspectos específicos del proyecto	Plazos insuficientes	43
	Incluir excepciones	19
	Reducir tamaño de las advertencias	12
	Reemplazar expresión “exceso” por “alto”	10
	Referir el tamaño de las advertencias al tamaño de la etiqueta	5
	No restringir donaciones	5
Efectos económicos negativos	Daño al comercio internacional	29
	Elevado costo de implementación	17
	Reducción de ventas	14

MERCOSUR, Mercado Común del Sur; OPS; Organización Panamericana de la Salud.

***Fuente:*** elaboración propia a partir del análisis de los comentarios recibidos en la consulta pública. Los porcentajes se calcularon con base en un total de 42 comentarios.

### Violación de compromisos internacionales

La mayoría de los comentarios recibidos hicieron referencia a que el proyecto de decreto crearía obstáculos técnicos al comercio, violando los compromisos internacionales asumidos por el país en los acuerdos resultantes de la Ronda de Negociaciones Comerciales Multilaterales (21). Estos argumentos han sido elevados a la OMC debido a la implementación obligatoria de rotulación nutricional frontal en Chile, Ecuador, Indonesia, Perú y Tailandia (22).

Si bien el Acuerdo sobre Obstáculos Técnicos al Comercio tiene por objeto garantizar que los reglamentos técnicos y las normas no constituyan trabas innecesarias al comercio internacional, reconoce que no debe impedirse a ningún país adoptar las medidas necesarias para proteger la vida y la salud y prevenir prácticas que puedan inducir las personas a error (21). En este sentido, cabe destacar que la protección de la salud tiene prioridad sobre las preocupaciones que atanen al comercio (23).

Algunos de los comentarios de la industria hicieron referencia a la ausencia de especificaciones sobre etiquetado nutricional frontal en el *Codex Alimentarius*, principal referencia de directrices, estándares y códigos internacional en materia de alimentos. Sin embargo, esto no impide que un país implemente una política pública y normas obligatorias en el marco de su derecho para determinar el nivel de protección que considere adecuado para alcanzar la protección de la vida y la salud de su población.

El proyecto de decreto fue considerado por la industria como incompatible con la normativa MERCOSUR (cuadro 1), la cual establece la obligatoriedad de declarar determinados contenidos nutricionales y la forma como se presenta esa información (24). Sin embargo, dicha normativa no constituye una limitante para que los Estados Parte puedan establecer reglamentación legítima para proteger la vida y la salud de sus poblaciones. La ausencia de parámetros de armonización regionales sobre etiquetado nutricional frontal legitima la aprobación del decreto. Además, el avance de uno de los países del bloque en la implementación de rotulado nutricional frontal facilita futuras armonizaciones en un bloque subregional, ya que la experiencia de ese país que tomó la acción permitiría informar al proceso de armonización. Por lo contrario, la armonización subregional sobre políticas con la cuales ninguno de los países tiene experiencia es mucho más difícil.

### Críticas al sistema de advertencias

La gran mayoría de los comentarios cuestionaron la selección del sistema de advertencias nutricionales para el etiquetado nutricional frontal, con el argumento de que estigmatiza alimentos, con el consiguiente miedo y confusión en los consumidores (cuadro 1). La industria destacó en sus comentarios que este sistema de rotulación nutricional posee un enfoque negativo hacia alimentos individuales y afirmó que ningún alimento es “malo” por sí mismo. Algunos de los comentarios denotaron que el sistema de advertencias es percibido como una prohibición hacia la población debido a la utilización de un octágono negro asociado con la señal de tránsito de “Pare”. Este argumento concuerda con el hecho de que la industria alimentaria se ha opuesto a sistemas de etiquetado nutricional que explicitan que un alimento posee una composición nutricional desbalanceada o alto contenido de nutrientes asociados con enfermedades no transmisibles (12). En este sentido, se ha observado un sesgo en la industria a destacar aspectos positivos de sus productos, tal como evidencia la utilización de alegaciones nutricionales en productos con elevado contenido de azúcares, grasas o sodio (25), o la utilización sesgada de sistemas resumen de etiquetado nutricional frontal (26). Es importante destacar que ninguno de los argumentos de la industria ha sido identificado al analizar la percepción de ciudadanos uruguayos sobre la rotulación nutricional frontal basada en advertencias (27).

Uno de los argumentos más mencionados en contra del sistema de advertencias fue que contraviene las disposiciones del *Codex Alimentarius* (2), en particular la disposición 3.5 sobre declaraciones de propiedades, la cual establece que deben prohibirse declaraciones que “pueden suscitar dudas sobre la inocuidad de alimentos análogos, o puedan suscitar o provocar miedo en el consumidor”. Este argumento es sacado de contexto, ya que la disposición 3.5 se aplica a “declaraciones de propiedades” que realiza la industria de manera opcional, o sea cualquier “descripción que afirme, sugiera o presuponga que un alimento tiene características especiales por su origen, propiedades nutritivas, naturaleza, producción, elaboración, composición u otra cualidad cualquiera” (2). Dicha disposición no se aplica al etiquetado frontal de advertencia prescripto por un Estado en protección de la vida y la salud. Además, no existe evidencia de que informar a los consumidores que un alimento tiene contenido excesivo de nutrientes pueda suscitar dudas sobre la inocuidad de los alimentos, o suscitar o provocar miedo en el consumidor (27). Por último, es importante destacar que el *Codex Alimentarius* establece “directrices voluntarias” que no impiden a los países tomar acciones más restrictivas si son necesarias para permitir decisiones informadas o mejorar la alimentación y salud de sus poblaciones.

La industria también alegó falta de evidencia científica para la selección del sistema de advertencias y sugirió la utilización de otros sistemas de etiquetado, tales como el sistema basado en la ingesta diaria recomendada (GDA, por sus siglas en inglés), el sistema semáforo o el Nutriscore. La selección del sistema de advertencias se basó en el objetivo regulatorio y en la evidencia científica generada a nivel nacional, que es consistente con la internacional y que permitió concluir que las advertencias poseen ventajas sobre otros sistemas en términos de su capacidad de captar la atención de los consumidores, de modificar la percepción de saludable de alimentos con excesivo contenido de azúcares, grasas, grasas saturadas y sodio y de motivar elecciones más saludables (7-9). Además, el resultado de un estudio cualitativo realizado durante el diseño del proyecto de decreto permitió comprobar una interpretación correcta de la información incluida en las advertencias nutricionales, sin verificar que este sistema pudiera generar miedo o dudas sobre la inocuidad y seguridad de los alimentos que la contienen (27).

### Discriminación de alimentos industrializados envasados

La mayoría de los comentarios hicieron referencia a que el proyecto de decreto de etiquetado nutricional frontal es discriminatorio al aplicarse solo a alimentos industrializados envasados (cuadro 1). En particular, la industria argumentó que el proyecto posee un enfoque negativo hacia los alimentos industrializados, lo que exacerba su papel en el aseguramiento de la inocuidad alimentaria y en la disponibilidad de alimentos en el contexto de una creciente población urbana. Además, se destacó la fortificación de nutrientes en alimentos industrializados como herramienta para prevenir deficiencias nutricionales vinculadas con diversas enfermedades. Estos argumentos no reconocen que un elevado porcentaje de los alimentos industrializados poseen un contenido excesivo de azúcares, sodio, grasas totales, grasas saturadas y grasas trans (14), los cuales están asociados a los factores que más causan muertes y pérdida de años de vida saludable (28). Por otra parte, cabe destacar que el decreto no hace énfasis en el procesamiento por sí mismo, sino en el agregado de nutrientes asociados con enfermedades no transmisibles. La decisión de considerar solo los productos alimenticios envasados con agregado de azúcares, grasas, grasas saturadas y sodio radica en la falta de evidencia científica sustantiva que determine que el consumo de alimentos naturales sin adición de nutrientes críticos tenga efectos adversos para la salud, contrario a la evidencia que existe para aquellos en que sí se han añadido. Por otro lado, de acuerdo con la alimentaria para la población uruguaya (29), no se recomiendan productos con agregado de esos nutrientes como parte de una alimentación saludable, y desplazan el consumo de los alimentos recomendados (30). Para cada uno de los nutrientes críticos considerados, la evidencia científica demuestra diferencias entre los alimentos que los contienen en forma natural y aquellos a los que se les han adicionado (30). En este sentido, cabe destacar que varios países recomiendan en sus guías alimentarias la reducción del consumo de alimentos con agregado de azúcares y sodio (31, 32) y que las recomendaciones de la Organización Panamericana de la Salud (OPS) indican que los alimentos naturales no deben incluir rótulo nutricional frontal que indique exceso de azúcares, grasas y sodio (14).

Además, se hizo referencia a que los alimentos no envasados disponibles en restaurantes, rotiserías y panaderías no serán rotulados a pesar de que su composición nutricional puede ser similar a la de los alimentos industrializados envasados. Algunos de los comentarios alegaron falta de evidencia sobre el rol de los alimentos industrializados envasados en la ingesta total de nutrientes asociados con enfermedades no transmisibles en el Uruguay. Este tipo de cuestionamientos sobre los alimentos que deben llevar etiquetado frontal se ha observado también en las discusiones de la OMC (33). Sin embargo, es abrumadora la evidencia que demuestra que dietas basadas en una mayor proporción de productos alimenticios procesados y ultraprocesados con agregado de azúcares, grasas y sodio, se asocian con un peor perfil nutricional (34), sobre todo en el caso de América Latina (30).

### Críticas al modelo de perfil de nutrientes

El modelo de perfil de nutrientes de la OPS seleccionado para la identificación de alimentos con excesivo contenido de azúcares, grasas, grasas saturadas y sodio fue muy cuestionado por la industria. Los comentarios argumentaron que el modelo carece de justificación científica al extrapolar las recomendaciones de la OMS de ingesta de nutrientes en la dieta a productos alimenticios individuales. En otras instancias, el modelo de perfil de nutrientes seleccionado para la implementación de sistemas de etiquetado nutricional frontal también ha sido cuestionado (22). El Modelo de Perfil de Nutrientes de la OPS, que fue presentado y discutido con todos los ministros de salud de América, tiene base en la evidencia del impacto de la ingesta excesiva de nutrientes críticos en la salud humana y ha sido sometido a los rigurosos procesos de revisión que se utilizan para establecer directrices y recomendaciones de ingesta de la OMS (35). El modelo se ajusta a los requerimientos energéticos de distintos rangos de edad, evitando exponer a los niños a criterios de ingesta definidos para adultos, y permite identificar productos que desequilibran la proporción de energía proveniente de azúcares y grasas, así como los productos que contribuyen proporcionalmente más al aporte de sodio establecido en las recomendaciones de la OMS. Por otro lado, el consumo de productos que cumplen con los criterios establecidos en el Modelo de Perfil de Nutrientes de la OPS no causa un incumplimiento de las metas de ingesta de nutrientes recomendadas por la OMS. Uruguay evitó la selección de un criterio arbitrario y se seleccionó el modelo de perfil de nutrientes desarrollado por la OPS, organismo internacional especializado en temáticas de salud pública. La industria afirmó que el modelo de perfil de nutrientes es sumamente estricto y que llevaría a que más del 80% de los productos presentaran al menos una advertencia frontal en su envase, sin estimular la reformulación de los productos comercializados en el mercado uruguayo, lo que parecería no tener una justificación adecuada.

Una de las críticas más frecuentes al modelo fue la consideración de límites basados en el porcentaje de calorías correspondiente a azúcares, grasas y grasas saturadas. La industria argumentó que los límites deberían tener en cuenta la porción de consumo y en varios casos se indicó que deberían ser diferenciales por categorías. Además, se indicó que sería preferible la consideración de límites absolutos por porción o 100 g de alimento. La base de cálculo propuesta en el modelo de perfil de nutrientes tiene la ventaja de utilizar una misma base de comparación para cualquier tipo de producto sin asumir una ingesta calórica diaria determinada, la cual varía de acuerdo con los requerimientos individuales.

En el caso de los nutrientes intrínsecos, se recibieron comentarios contrarios. Algunas industrias indicaron como una incoherencia del proyecto el hecho de no incluir etiquetado frontal en alimentos sin agregado de azúcares, grasas o sodio, mientras que en el caso de las industrias lácteas se solicitó la no consideración de la lactosa y la grasa intrínseca de estos productos en los límites. Otros comentarios relacionados con el perfil de nutrientes sugirieron excluir las grasas totales del modelo, la modificación del límite de sodio en productos bajos en calorías y la inclusión de calorías en el modelo (cuadro 1).

### Otras críticas a aspectos específicos del proyecto

Se recibieron críticas a aspectos específicos del proyecto, de las cuales la mencionada con mayor frecuencia fue la falta de tiempo para la implementación de la política (cuadro 1). Las industrias argumentaron que el plazo de un año proyectado en el proyecto de decreto era insuficiente y solicitaron una extensión que varió entre los 24 y 48 meses. Comentarios similares se han registrado en las discusiones de la OMC sobre otras reglamentaciones de etiquetado frontal (22).

Otras críticas mencionadas con menor frecuencia estuvieron vinculadas con la inclusión de excepciones a productos específicos (p.ej., alimentos para deportistas), la reducción del tamaño de las advertencias en los envases, reemplazar la palabra “exceso” por “alto” en el diseño del etiquetado, referir el tamano de las advertencias al de la etiqueta en lugar del tamaño del envase y la eliminación del artículo referido a la restricción de donaciones de alimentos con advertencias frontales al Estado.

### Efectos económicos negativos

Por último, los comentarios de la industria también incluyeron algunos sobre el impacto económico negativo del decreto de rotulación frontal, afirmando que ocasionaría daños al comercio internacional, aumento de precios debido a su elevado costo de implementación y reducción de ventas en el mercado uruguayo. Sin embargo, la industria no presentó estudio alguno que comprobara tales alegaciones.

### Limitaciones y fortalezas del trabajo

La principal limitación del trabajo es el hecho de que solo se analizaron los comentarios enviados en la consulta pública realizada por el gobierno uruguayo antes de la implementación de las advertencias nutricionales. Dichos comentarios no pueden ser considerados representativos de la postura de todas las industrias de alimentos. En lo que respecta a las fortalezas, debe destacarse que se analizaron comentarios enviados de manera espontánea por la industria en el marco de un proceso regulatorio, por lo que su contenido refleja los argumentos que podrían ser utilizados en actividades políticas corporativas para influir sobre políticas de gobierno.

## CONCLUSIONES

El análisis de los comentarios de la industria recibidos durante la consulta pública implementada durante el diseno del decreto de rotulación nutricional frontal de Uruguay permitió visualizar una fuerte oposición a la política y, en particular, al sistema de advertencias. La mayoría de los argumentos en contra de la política fueron similares a los registrados en otros procesos regulatorios y discusiones en la Organización Mundial del Comercio. Los resultados aportan insumos a gobiernos y tomadores de decisión que estén considerando la implementación de advertencias nutricionales para facilitar la identificación de productos alimenticios con excesiva cantidad de nutrientes asociados con enfermedades no transmisibles.

### Contribución de los autores.

Todos los autores concibieron el estudio original. GA e IB participaron del análisis de datos. GA, IB y FG escribieron una primera versión del manuscrito, a lo que el resto de los autores contribuyeron de forma significativa. Todos los autores revisaron y aprobaron la versión final.

### Financiamiento.

Los autores agradecen la financiación otorgada por el Espacio Interdisciplinario de la Universidad de la República.

### Declaración.

Las opiniones expresadas en este manuscrito son responsabilidad del autor y no reflejan necesariamente los criterios ni la política de la *RPSP/PAJPH* y/o de la OPS.
